# Comprehensive Analysis of Physicochemical, Functional, Thermal, and Morphological Properties of Microgreens from Different Botanical Sources

**DOI:** 10.1021/acsomega.3c03429

**Published:** 2023-08-03

**Authors:** Sanyukta, Dilpreet Singh Brar, Kirty Pant, Sawinder Kaur, Vikas Nanda, Gulzar Ahmad Nayik, Seema Ramniwas, Prasad Rasane, Sezai Ercisli

**Affiliations:** † Department of Food Engineering and Technology, Sant Longowal Institute of Engineering and Technology, Longowal, 148106 Sangrur, Punjab, India; ‡ Department of Food Science and Nutrition, Lovely Professional University, Phagwara 144001, Punjab, India; § Department of Food Science & Technology, Government Degree College Shopian, Shopian 192303, Jammu and Kashmir, India; ∥ University Centre for Research and Development, Chandigarh University, Gharuan, Mohali 140413, Punjab, India; ⊥ Department of Horticulture, Faculty of Agriculture, Ataturk University, 25240 Erzurum, Turkey; # HGF Agro, Ata Teknokent, TR-25240 Erzurum, Turkey

## Abstract

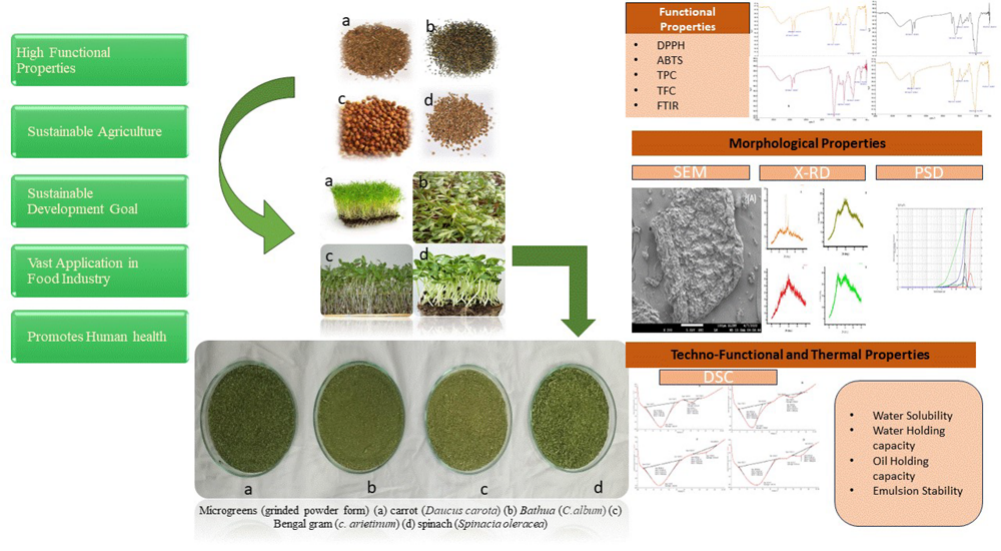

Due to the significant increase in global pollution and a corresponding decrease in agricultural land, there is a growing demand for sustainable modes of modern agriculture that can provide nutritious food. In this regard, microgreens are an excellent option as they are loaded with nutrients and can be grown in controlled environments using various vertical farming approaches. Microgreens are salad crops that mature within 15–20 days, and they have tender leaves with an abundant nutritive value. Therefore, this study aims to explore the physicochemical, techno-functional, functional, thermal, and morphological characteristics of four botanical varieties of microgreens, including carrot (*Daucus carota*), spinach (*Spinacia oleracea*), *bathua* (*Chenopodium album*), and Bengal gram (*Cicer arietinum*), which are known for their exceptional nutritional benefits. Among the four botanical varieties of microgreens studied, *bathua* microgreens demonstrated the highest protein content (3.40%), water holding capacity (1.58 g/g), emulsion activity (56.37%), and emulsion stability (53.72%). On the other hand, Bengal gram microgreens had the highest total phenolic content (32.2 mg GAE/g), total flavonoid content (7.57 mg QE/100 g), and DPPH activity (90.60%). Fourier transform infrared spectroscopy analysis of all microgreens revealed the presence of alkanes, amines, and alcohols. Moreover, X-ray diffraction analysis indicated low crystallinity and high amorphousness in the microgreens. Particle size analysis showed that the median, modal, and mean sizes of the microgreens ranged from 110.327 to 952.393, 331.06 to 857.773, and 97.567 to 406.037 μm, respectively. As per the observations of the results, specific types of microgreens can be utilized as an ingredient in food processing industry, including bakery, confectionery, and more, making them a promising nutritive additive for consumers. This study sheds light on various food-based analytical parameters and offers a foundation for future research to fully harness the potential of microgreens as a novel and sustainable food source, benefiting both the industry and consumers alike.

## 1 Introduction

Rapid urbanization and population growth have heightened the need for nutrient-rich food in cities, and many individuals seek natural and nutritious solutions to address modern health challenges.^1^ To meet these demands sustainably, modern solutions such as vertical farming and cultivation of short-duration crops are increasingly being adopted.^2^ Microgreens, which are short-duration crops, are particularly promising, as they can be efficiently grown in vertical farms for large-scale production or in home kitchen gardens due to their nutrient-rich nature, containing vitamins, minerals, and other bioactive components.^3^ Adopting such solutions can offer a sustainable response to present-day concerns surrounding nutrition and food security.^4^

Microgreens are a salad crop harvested within 10–20 days of seedling emergence, featuring young and tender leaves with two fully grown cotyledon leaves and the first pair of true leaves either appearing or partially developed.^5^ These petite greens come in a variety of colors, textures, and flavors and are typically 2.5–6 cm tall, smaller than baby greens.^6,7^ Unlike sprouts, microgreens have already developed their first true leaves.^8,6^ The most common species of microgreens belong to families such as *Amaranthceae*, *Apiaceae*, *Asteraceae*, *Chenopodiaceae*, *Brassicaceae*, *Lamiaceae*, *Cucurbitaceae*, and *Amarillydaceae*.^5^ Microgreens are a highly nutritious addition to any diet due to their rich content of vitamins, minerals, and antioxidants. Compared to seeds or mature plants, microgreens are abundant in simple sugars, free amino acids, fatty acids, vitamins, minerals, and phytochemicals such as ascorbic acid, beta-carotene, and alpha tocopherol. Additionally, microgreens contain lower levels of antinutrients,^1,9^ making them an ideal source of essential nutrients. Previous studies, including research by Treadwell et al.^8^ and Sharma et al.,^10^ have emphasized the importance of microgreens in promoting human health.

In this study, we aim to develop microgreen powder from carrot (*Daucus carota*), spinach (*Spinacia oleracea*), *bathua* (*Chenopodium album*), and Bengal gram (*Cicer arietinum*) with versatile food applications, which can be determined by a comprehensive analysis of their physicochemical, techno-functional, functional, thermal, and morphological properties. This approach will enable us to understand the impact of different botanical families on the properties and nutritional value of microgreens and identify the best microgreens for the development of safe and nutritious food products.

To the best of our knowledge, no previous research work has been reported on the comparative analysis of physicochemical, techno-functional, functional, thermal, and morphological properties of microgreens from carrot (*Daucus carota*), spinach (*Spinacia oleracea*), *bathua* (*Chenopodium album*), and Bengal gram (*Cicer arietinum*) in the same season.

## 2 Materials and Methods

### 2.1 Chemicals, Standards, and Reagents

All the chemicals were of good grade and were purchased from HiMedia Leading BioScience Company. The chemicals and reagents, which are used during the project work are as follows: sodium hydroxide, sodium chloride, sodium acetate, methanol, petroleum ether, Folin–Ciocalteu reagent, sodium hydroxide, sodium carbonate, sodium nitrite, and aluminum chloride, which were obtained from Loba Chemie (Mumbai, India). Gallic acid, quercetin, and 2,2-diphenyl-1-picrylhydrazyl (DPPH) were obtained from Sigma-Aldrich (St. Louis, Missouri, USA).

### 2.2 Sample Collection and Preparation

**Figure 1:**
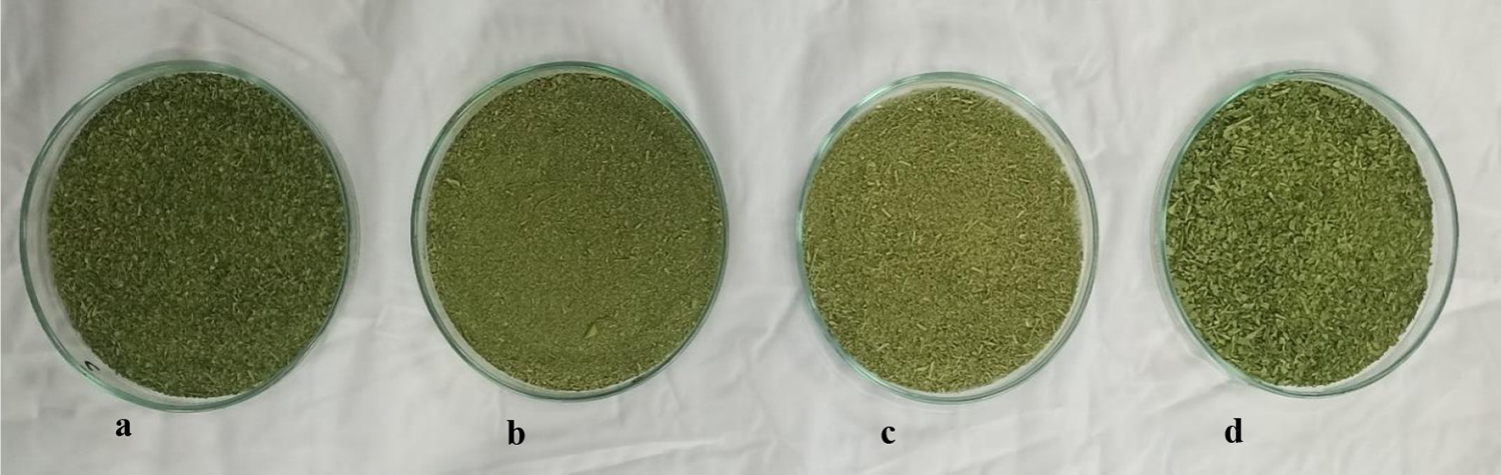
Microgreens (ground powder form) of (a) carrot (*D. carota*), (b) *bathua* (*C. album*), (c) Bengal gram (*C. arietinum*), and (d) spinach (*S. oleracea*).

The four microgreens spinach (*Spinacia oleracea*), *bathua* (*Chenopodium album*), carrot (*Daucus carota*), and Bengal gram (*Cicer arietinum*) were obtained from the local fields of Longowal, Punjab. These microgreens were grown in growth chambers at 25 °C and harvested on 14–16 days when two leaflets were visible on the stalk; a white fluorescent light tube was used for providing light for 12 h a day. The harvested samples were cleaned to remove any extraneous matter and dirt. These were then washed with deionized water, dried in a tray dryer at 55 °C for 8–9 h, and stored at room temperature until further analysis, as shown in Figure 1.

### 2.3 Physicochemical Analysis

The microgreen samples were analyzed for moisture, protein, ash, crude fat, and fiber content in triplicates according to AOAC, respectively.^11^

#### 2.3.1 Color

A color spectrophotometer (CH-8105, Regensdorf, Switzerland) was used to determine the color of the microgreens. The chroma (c*) values and hue angle (*h***°**) were observed.

### 2.4 Functional Properties

#### 2.4.1 Total Phenolics and Flavonoids

To estimate total phenolics and flavonoids, methanolic extracts of the microgreen samples (1 g) were prepared with 50% methanol. The Folin–Ciocalteu method with some modifications was used.^12^ The absorbance was measured at 760 nm, and by using gallic acid solutions of different concentrations between 0 and 100 mg/mL, a standard curve was prepared, and the results were expressed as mg of gallic acid equivalents (mg GAE/100 g) of extract. The determination of total flavonoids was done using the method given in ref (13). The absorbance was measured at 510 nm, and as standard quercetin was used, the results were expressed as mg of quercetin equivalents/100 gm of extracts.

#### 2.4.2 DPPH Radical Scavenging Activity

The DPPH radical scavenging activity was measured by the method as described in ref (14) using a spectrophotometer (Hach Lange DR6000 UV–VIS) at 517 nm absorbance. It was calculated by the formula:
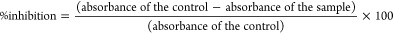


#### 2.4.3 2,2′-Azino-bis(3-ethylbenzothiazoline-6-sulfonic acid) (ABTS^+^)

It was determined using the methodology described by Re et al.^15^ To prepare 7 mM ABTS solution, ABTS salt was dissolved in water (16 h), and the solution was further diluted with methanol to get an absorbance of 0.700 at 734 nm. Moreover, to form ABTS^+•^ radicals of concentration 2.45 mM, potassium persulfate (7 mM) was added to ABTS solution. The prepared mixture was then agitated for 1 min and was allowed to incubate for 10 min under dark and ambient conditions. Absorbance was measured at 734 nm. The microgreen extracts (100 μL) were mixed with 3.9 mL of the ABTS reagent and absorbance was measured at 517 nm after incubating it for 20 min.^15^ The ABTS radical scavenging activity was determined as follows:
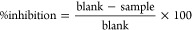


### 2.5 Techno-Functional Properties

#### 2.5.1 Water Solubility

The powdered sample (0.2 g) was mixed with 20 mL of distilled water, and it was then centrifuged at 4500 rpm for 10 min. After centrifugation, the supernatant (5 mL) was then dried in an oven at 105 °C until a constant weight was achieved. The mass of the sample obtained after drying was used to determine the solubility.^16^
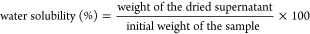


#### 2.5.2 Water Holding Capacity

The powdered sample (2.5 g) was taken in centrifuge tubes. It was well mixed with 10 mL of distilled water and allowed to stand for 30 min at room temperature at 22 ± 2 °C. After centrifugation at 1200 *g* for 30 min, the supernatant was decanted carefully, and the new mass of the sample was recorded.^16^

#### 2.5.3 Oil Holding Capacity

The powdered sample (0.5 g) was mixed with 6 mL of refined soya oil in a preweighed centrifuge tube. The suspension was held at 25 °C for 30 min at 3000 *g*. The tube was inverted for 25 min after decanting the separated oil layer to drain the excess oil before weighing.^17^

#### 2.5.4 Emulsion Activity and Stability (EA and ES)

The emulsion activity was determined as per the method described by Okezie and Bello,^18^ with some modifications. One gram of the sample was taken and mixed with 12.5 mL of distilled water, and 12.5 mL of soy oil was added slowly and mixed after thorough dispersion. It was then centrifuged at 3000 rpm for 5 min, and the volume of oil separated from the sample was recorded. The ratio of the height of the emulsion to the total height was considered as the emulsion activity (%). The emulsion stability of the samples was determined by heating the fully prepared emulsion at 80 °C for 30 min and then kept in cold water for 15 min. The emulsion was then centrifuged at 1300 *g* for 5 min, and the emulsion stability was determined by:
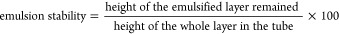


#### 2.5.5 Foaming Capacity and Stability (%) (FC and FS)

The foaming capacity of the various microgreens was determined as per the method given by Okezie and Bello;^18^ 2 g of the sample was whipped for 5 min with 100 mL of distilled water in a waring blender. It was then poured into a 250 mL measuring cylinder. The foaming capacity was calculated as:
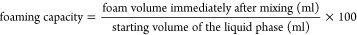


Foaming stability was calculated as the change in the volume of the foam after 1 h of mixing.
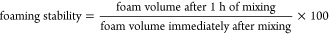


### 2.6 Morphological Characteristics

#### 2.6.1 Scanning Electron Microscopy

The morphology of samples was determined by scanning electron microscopy (SEM, JSM-6510 LV SEM, JEOL, Ltd., Tokyo, Japan). On SEM aluminum stubs, the microgreen powdered sample that is sputter-coated with platinum is dusted. By employing a high voltage of 10 KV and at a magnification of 2000× micro images of the sample were captured.

#### 2.6.2 X-ray Diffraction

An X-ray diffractometer (PANalytical X’ pert PRO MRD, Almelo, the Netherlands) was used to determine the crystalline or amorphous nature of the microgreens. At angles ranging between 10° and 50° (2θ) with a step size of 0.02°, the microgreen samples were evaluated with a rate of 1 step/s.

#### 2.6.3 Fourier Transform Infrared Spectroscopy

A Fourier transform infrared (FTIR) spectrophotometer (Spectrum TWO LiTa, Llantrisant, UK) with an attenuated total reflection accessory was used to obtain spectra of various microgreens. On the ZnSe crystal plate, sample dust was placed, and at the absorbance range of 4000 to 400 cm^–1^ the FTIR spectrum was determined with 1 cm^–1^ resolution.

#### 2.6.4 Particle Size Distribution

For measuring the particle size of the microgreens, a Shimadzu particle size analyzer (Shimadzu SALD-2300 WingSALD II: Version 3.1.0) was used. The particle size distribution (PSD) of the samples was measured within 0.017–2500 μm by laser diffraction and the laser scattering intensity pattern at a wavelength of 720 nm; 0.5 g of the powdered sample was dispersed in water before filling into a cuvette. Then, the readings (mean, median, and modal) were taken during successive two to five trials.

### 2.7 Thermal Properties

#### 2.7.1 Differential Scanning Calorimetry

Differential scanning calorimetry (DSC, PerkinElmer DSC 4000, serial no. N520-0112) was used to determine the thermal properties of the microgreens. To monitor and regulate the temperature up to −20 °C, a refrigerated cooling system (RCS) was connected to the system. The sample about 10–20 mg was loaded in aluminum pans, sealed hermetically, and further scanned over a temperature of −20 to 200 °C at 10 °C/min. An empty aluminum pan, which was hermetically sealed, was used as a reference. At a rate of 50 mL/min, nitrogen was employed as a purge gas. The results were obtained using TRIOS software v4.2.1.36612 (TA Instruments), and the values for (*T*_o_) onset temperature, (*T*_p_) peak temperature, (*T*_e_) end set temperature, and (ΔHU) enthalpy change were determined.^19^

### 2.8 Statistical Analysis

For the analysis of data, ANOVA and Duncan post-hoc tests were applied using the Statistical Package for Social Sciences (SPSS) version 16.0 (Chicago, USA).

## 3 Results and Discussions

### 3.1 Physicochemical Analysis

The moisture content of the microgreens is influenced by various factors such as climatic conditions, processing techniques, and postharvest storage conditions. Our findings indicate that the moisture content of all the microgreens examined in this study exhibited a statistically significant difference (*p* < 0.05) (Table 1). Additionally, we observed that the moisture content ranged from 80.83% in Bengal gram to 92.63% in spinach, with spinach exhibiting the highest moisture content and Bengal gram exhibiting the lowest.

**Table 1:** Physicochemical Composition of Microgreens^a^

parameter	spinach	carrot	*bathua*	Bengal gram
moisture (%)	92.63 ± 0.54^a^	83.46 ± 0.93^c^	89.56 ± 0.47^b^	80.83 ± 0.28^d^
crude fat (%)	0.43 ± 0.04^b^	0.31 ± 0.10^cd^	0.53 ± 0.01^a^	0.33 ± 0.12^c^
protein (%)	2.56 ± 0.14^bc^	2.43 ± 0.06^b^	3.40 ± 0.15^a^	2.6 ± 0.07^bc^
crude fiber (%)	1.16 ± 0.12^bc^	2.40 ± 0.14^a^	1.11 ± 0.06^d^	1.31 ± 0.07^b^
ash (%)	1.21 ± 0.01^c^	1.36 ± 0.04^a^	1.32 ± 0.01^ab^	1.20 ± 0.02^bc^
_L_*	49.31 ± 0.02^cd^	48.66 ± 0.46^c^	51.03 ± 0.33^b^	53.57 ± 0.46^a^
_a_*	–5.92 ± 0.08^d^	–5.34 ± 0.02^c^	–4.86 ± 0.01^b^	–4.49 ± 0.09^a^
_b_*	17.23 ± 0.01^b^	13.05 ± 0.09^d^	15.04 ± 0.08^c^	17.77 ± 0.01^a^
_c_*	18.23 ± 0.08^ab^	14.12 ± 0.08^c^	15.79 ± 0.09^b^	18.30 ± 0.08^a^
*_h_*°	108.98 ± 0.08^b^	112.37 ± 0.08^a^	107.95 ± 0.01^c^	104.19 ± 0.01^d^

a Values are means ± SD of triplicate analysis. Means with different letters in the same row indicate significant differences at *p <* 0.05.

Based on the USDA food composition databases,^20^ the fat content of microgreens is considered negligible and is similar to the average values observed in mature leaves. This study reported that the microgreens of spinach had the highest fat content (0.43%), while the lowest value of fat content was observed in carrot microgreens (0.31%). Among the families of microgreens studied, the Chenopodiaceae family (*bathua*) exhibited slightly higher protein content (3.40%) than *Fabaceae* (Bengal gram) (2.6%), *Amaranthaceae* (spinach) (2.56%), and *Apiaceae* (carrot) (2.43%). Additionally, our findings showed that the protein content in spinach was slightly higher than that reported in a previous study by Ghoora et al.^1^ However, the protein content of Bengal gram microgreens is less in comparison to the investigation done by Kaur et al.^21^ Carrot microgreens exhibited the highest dietary fiber content (2.4%) compared to the *Amaranthaceae* and *Fabaceae* families. Microgreens obtained from carrot exhibited the highest ash content; however, the maximum value observed did not exceed 1.36%. These variations in the composition of microgreens are due to the difference in the cultivation areas, climatic conditions, variety, and nutrient media. Moreover, the illumination intensity, uniform supply of specific supplement in nutrient media, and insect or pest attack (disease) also influence the nutritive composition of microgreens.^22^

The L* value, representing the degree of lightness, was the highest in Bengal gram (53.57), followed by *bathua*, spinach, and carrot microgreens. The microgreens displayed a negative (−a*) value, indicating the presence of green color, which ranged between −5.92 and −4.49, respectively. On the other hand, Bengal gram exhibited a higher b* value of 17.77, indicating the dominance of yellow color, followed by spinach (17.23), *bathua* (15.04), and carrot (13.05). Our study showed that the negative (−a*) and positive (b*) values placed all four microgreens in the greenish-yellowish region of the LAB space (Table 1). The hue angles for the microgreens ranged between 104.19° and 112.37°, indicating that the color varies from green to yellow. Moreover, the chroma values were observed to be in the range of 14.12 to 18.23. Therefore, it can be concluded that all the microgreens were in the greenish to yellowish color range, and all the values exhibited significant variations (*p* < 0.05), as the green-yellow color of microgreens is due to the chlorophyll pigment present in the tender leaves.^1^ The results of color in our study are evidence of factors, which influence the color of microgreens. The factors are botanical origin, varietal difference, exposure to sunlight, storage conditions, etc.^23,24^

### 3.2 Functional Properties

#### 3.2.1 Total Phenolic Content and Total Flavonoid Content

In this study, the total phenolic content (TPC) and total flavonoid content (TFC) were determined to be significant (*p* < 0.05), as shown in Table 2. Several internal and external factors, such as growing conditions, maturity at harvest, sample preparation, and species, can affect the phenolic content of microgreens.^25^ The TPC values for the microgreens ranged from 15.1 to 32.2 mg GAE/100 g, and the highest value of TPC was reported in Bengal gram and lowest in spinach, whereas a similar value of TPC was observed in carrot (28.30 mg GAE/100 g) and *bathua* (28.80 mg GAE/100 g). Similar results for TPC content in spinach microgreens were observed.^28^ Furthermore, TFC content in value ranged between 1.90 mg QE/100 g and 7.57 mg QE/100 g, lowest in spinach and highest in Bengal gram. The TPC and TFC values of carrot microgreens are in accordance with the results observed in a study conducted by Ghoora et al.^1^

**Table 2:** Determination of Functional Activity Based on Bioactive Compounds of Microgreens^a^

parameters	spinach	carrot	*bathua*	Bengal gram
TPC (mg GAE/100 g)	15.10 ± 0.16^c^	28.30 ± 0.32^b^	28.80 ± 0.08^b^	32.20 ± 0.08^a^
TFC (mg QE/100 g)	1.90 ± 0.06^d^	5.48 ± 0.08^b^	4.77 ± 0.04^c^	7.57 ± 0.08^a^
DPPH (%)	46.30 ± 0.41^d^	89.54 ± 0.03^ab^	81.76 ± 0.08^c^	90.60 ± 0.81^a^
ABTS (%)	29.36 ± 0.03^d^	81.85 ± 0.04^a^	35.04 ± 0.04^b^	33.11 ± 0.08^c^

a Values are means ± SD of triplicate analysis. Means with different letters in the same row indicate significant differences at *p <* 0.05.

#### 3.2.2 DPPH (2,2-Diphenyl-1-picrylhydrazyl)

In DPPH, when the antioxidants present in the sample extract react with the DPPH radical, a hydrogen atom is donated and converted into a reduced form.^26^ Furthermore, the level of discoloration determines the radical scavenging potential during the reaction, which further implies that more antioxidants present in the sample will give a higher DPPH value. Moreover, the antioxidant activity is directly related to the TPC and TFC content, as similar trend was observed in the antioxidant activity of four microgreens. The DPPH values varied from 46.3 to 90.60%, as spinach had the lowest value, 46.3%, and Bengal gram had the highest, that is, 90.60%, whereas the antioxidant activity of carrot and *bathua* was 89.54 and 81.76%, respectively, which depicted significant differences (*p* < 0.05) (Table 2). The similar antioxidant activity in carrot microgreens was observed, which was around 90%.^1^ However, *bathua* microgreens had higher antioxidant activity than the *Bathua* flour that ranged between 14.10 and 20.33%.^27,28^

#### 3.2.3 ABTS (ABTS^+•^) Radical Scavenging Activity

In the current study, the ABTS salt and potassium persulfate were combined to generate ABTS free radicals, which were subsequently used to evaluate the antioxidant properties and efficacy of the test samples in scavenging or neutralizing the free radicals. The microgreens under investigation exhibited varying ABTS scavenging potentials (29.36 to 81.85%), with statistically significant differences (*p* < 0.05) observed among the samples. The ABTS free radical scavenging activities are presented in Table 2, with carrot exhibiting the highest scavenging potential (81.85%), followed by *bathua* (35.04%), Bengal gram (33.11%), and spinach (29.36%). Moreover, similar results of antioxidant activity with ABTS^+^ were observed in the carrot microgreen.^1^ The study conducted by Petropoulos et al.^29^ also reported that the antioxidant activity values of spinach analyzed by ABTS^+^ were in accordance to the observation of our results.

### 3.3 Techno-Functional Properties

In this study, the water solubility of spinach microgreens was found to be the highest among all samples (0.03%). The water holding capacity (WHC) of the microgreens ranged from 1.20 g H_2_O/g powder (carrot) to 1.58 g H_2_O/g powder (*bathua*), with statistically significant differences (*p* < 0.05) observed among the samples (Table 3). The higher WHC of *bathua* might be attributed to its higher protein content, as protein subunits have more water-binding sites.^30^ The oil holding capacity (OHC) of the microgreens varied from 1.6 to 3.74 g/g, with the maximum value observed for spinach microgreens and the minimum for *bathua* microgreens. This can be explained by the particle size, as a decrease in particle size leads to an increase in OHC.^31^ Emulsion activity and stability are influenced by factors such as molecular size, net charge, and molecular flexibility.^32^ The highest values for both emulsion activity and stability were observed in *bathua*, possibly due to the higher surface hydrophobicity of globulins compared to albumins. Furthermore, an increase in pH can increase the Coulombic interaction between neighboring droplets, leading to increased emulsion activity and stability.^33^ The foam capacity and stability varied significantly (*p* < 0.05), with the highest foam capacity observed in Bengal gram microgreens (33.7%) and the lowest in carrot microgreens (14.89%). Similarly, the foam stability was the highest in Bengal gram microgreens (98%) and the lowest in carrot microgreens (42.83%).

**Table 3:** Techno-Functional Properties of Microgreens^a^

parameters	spinach	carrot	*bathua*	Bengal gram
water holding capacity (WHC) (g/g)	1.23 ± 0.01^c^	1.20 ± 0.08^c^	1.58 ± 0.04^a^	1.31 ± 0.08^b^
solubility (%)	0.03 ± 0.08^a^	0.02 ± 0.08^b^	0.02 ± 0.08^b^	0.01 ± 0.04^c^
oil holding capacity (OHC) (g/g)	3.74 ± 0.08^a^	3.32 ± 0.08^b^	1.60 ± 0.16^d^	2.50 ± 0.08^c^
emulsion activity (EA) (%)	51.0 ± 0.70^b^	47.04 ± 0.82^c^	56.37 ± 0.82^a^	52.56 ± 0.42^b^
emulsion stability (ES) (%)	50.33 ± 0.47^b^	45.10 ± 0.03^c^	53.72 ± 0.13^a^	50.90 ± 0.48^b^
foaming capacity (FC) (%)	26.5 ± 0.08^b^	14.89 ± 0.29^d^	18.08 ± 0.04^c^	33.7 ± 0.43^a^
foaming stability (FS) (%)	52.23 ± 0.32^c^	42.83 ± 0.04^d^	88.23 ± 0.01^b^	98 ± 0.81^a^

a Values are means ± SD of triplicate analysis. Means with different letters in the same row indicate significant differences at *p <* 0.05.

### 3.4 Morphological Characteristics

#### 3.4.1 Scanning Electron Microscopy

**Figure 2:**
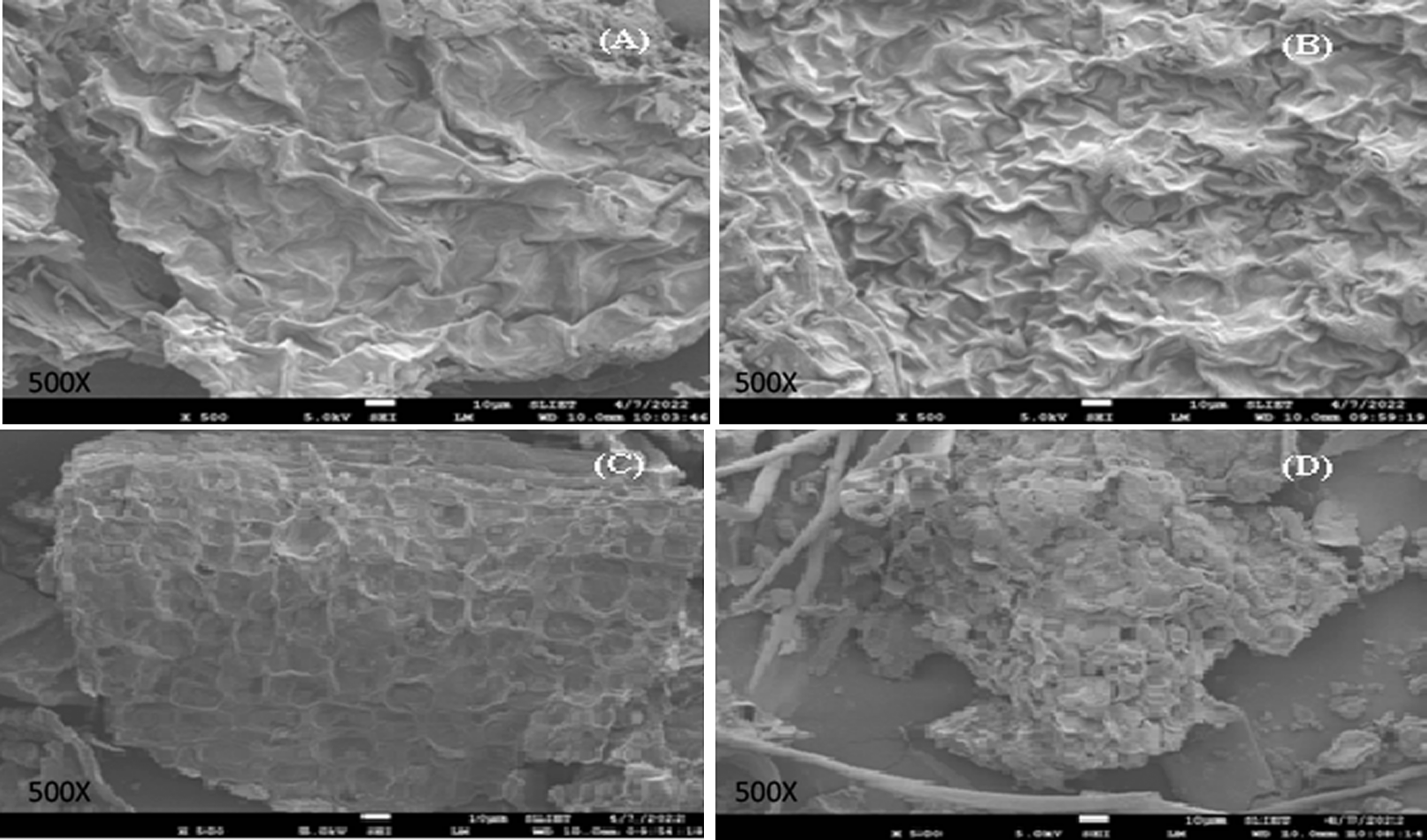
SEM (500×) images of (A) spinach, (B) carrot, (C) *bathua*, and (D) Bengal gram.

The morphological structures of the four microgreens were examined using SEM at 500× (Figure 2). SEM is a valuable tool for analyzing microstructures. In the present study, slight variations in the morphological structure of the selected microgreens (spinach, carrot, *bathua*, and Bengal gram) were observed. All the microgreen samples were examined at a magnification of 500×. The SEM images of spinach microgreens revealed a nonuniform, irregular pattern with slight loose folds on the surface (Figure 2A). The micrographs of carrot microgreens (Figure 2B) showed an uneven, rough surface with dense zigzag creases. The microgreens of *bathua* displayed a round structure with a few circular cavities and small depressions on the surface, along with some folds in the corners (Figure 2C). In contrast, the photomicrographs of the Bengal gram microgreens exhibited elongated stalklike structures, along with small clusters of uneven, asymmetric patterns on the surface (Figure 2D).

#### 3.4.2 X-ray Diffraction

**Figure 3:**
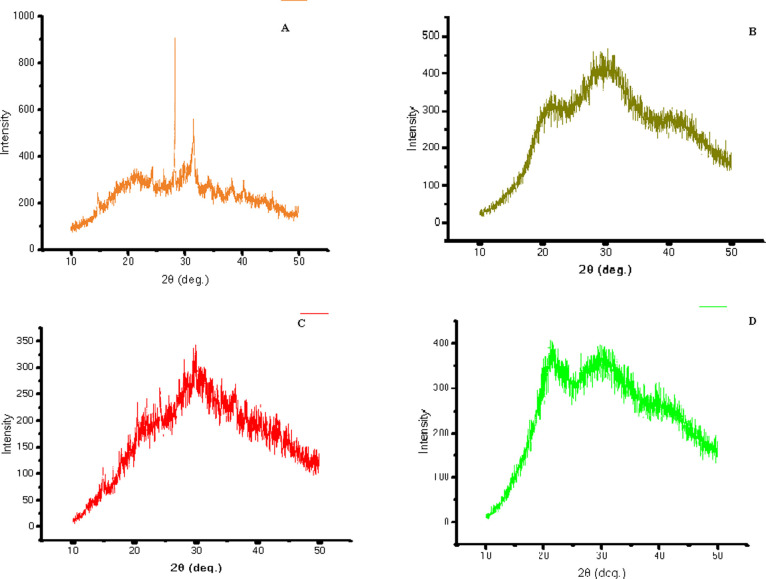
XRD representation of (A) spinach, (B) carrot, (C) *bathua*, and (D) Bengal gram.

In the present study, X-ray diffraction (XRD) patterns for microgreens are shown in Figure 3. XRD is an important tool for determining the degree of crystallinity and providing information about the presence and characteristics of crystalline constituents in a sample. The diffraction pattern of spinach microgreens (Figure 3A) showed a sharp peak at around 28° and 32°, indicating slight crystalline behavior induced by specific compounds in the sample. This may be due to the presence of starch, as the crystallinity of starch differs with the crystal size and amount of the region that is crystalline.^34^ In contrast, the diffraction pattern of carrot microgreens (Figure 3B) showed peaks starting from 10°, but the intensity and broadness of the peak increased from 20°, indicating a high degree of amorphous nature, which may be due to the presence of the amylose chain.^35^ The diffraction pattern of *bathua* microgreens (Figure 3C) showed an inverted “V” type graph with broader peaks compared to spinach and carrot microgreens, indicating a more amorphous nature of the product. The XRD of *bathua* flour also showed an “A” type diffraction pattern.^26^ The microgreens of Bengal gram (Figure 3D) showed an “M” type graph, with broad peaks in the region from around 21° to 50°, indicating the presence of nano-sized particles in the sample and revealing the amorphous characteristics of the sample. The size of the crystal depends on the diffraction intensity and angles, and if the diffraction angle is larger and intensity of diffraction is small, then the size of the crystal will also be small.^32^

#### 3.4.3 Fourier Transform Infrared Spectroscopy

**Figure 4:**
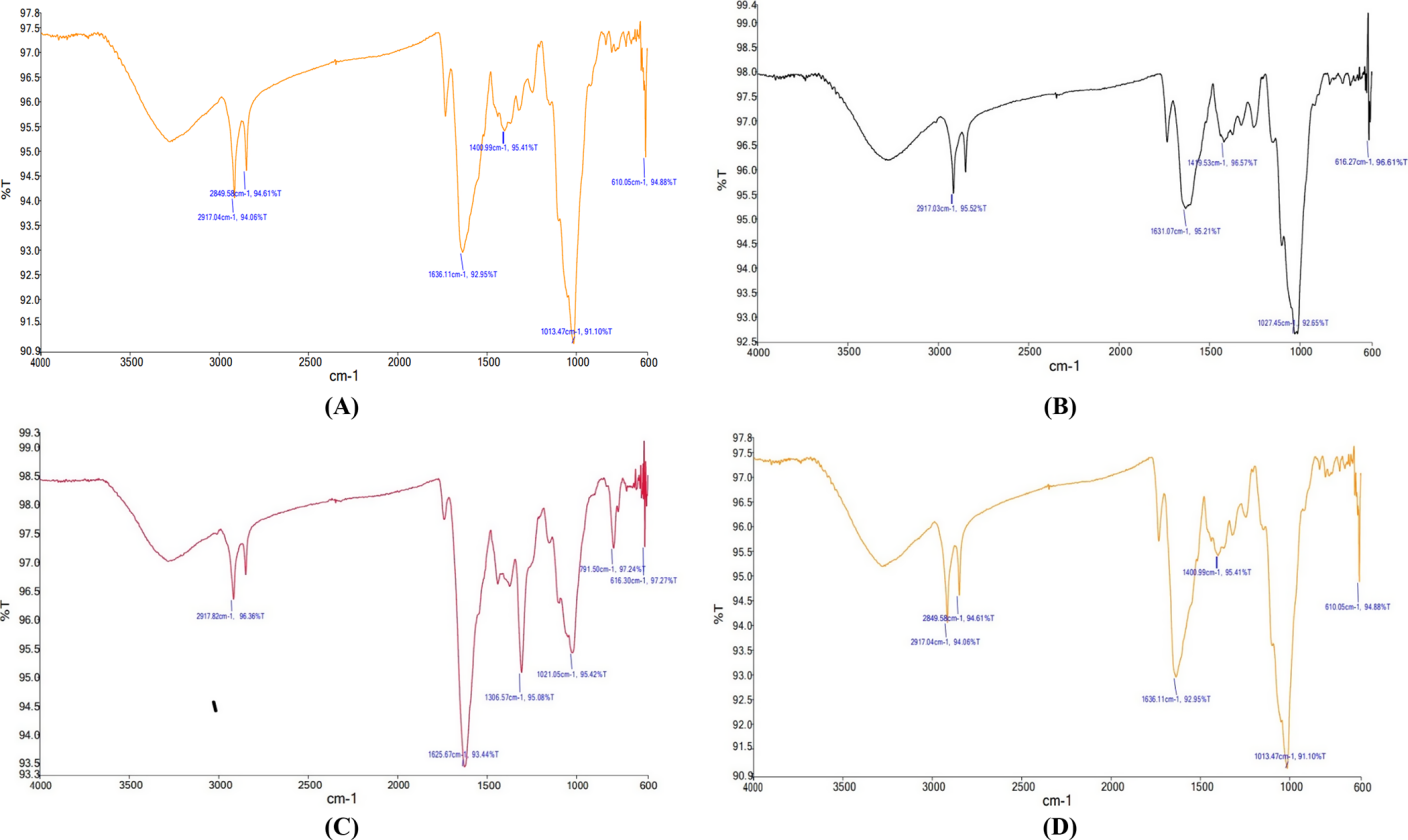
FTIR spectra of microgreens of (A) spinach, (B) carrot, (C) *bathua*, and (D) Bengal gram.

Four different microgreens were analyzed using FTIR spectroscopy to determine the functional groups present in the samples. The results were presented as peaks in the spectra, which are shown in Figure 4. The FTIR spectrum of spinach microgreens (Figure 4) showed peaks at 3280.27, 2917.31, 773.91, and 607.60 cm^–1^, which were attributed to alcohols (O–H stretching), alkanes (C–H stretching), alkenes (C=C stretching), phenols (O–H bending), amines (C–N stretching), and aliphatic bromo compounds (C–Br stretching).^36−38^ The peaks in the region between 1500 and 1000 cm^–1^ indicated the presence of polyphenols and proteins. Carrot microgreens (Figure 4) showed peaks in the region between 2917.03 and 616.27 cm^–1^. The peak at 2917.03 cm^–1^ was attributed to alkanes (C–H stretching), and the region between 1400 and 1000 cm^–1^ showed the presence of alcohols (O–H bending) and amines (C–N stretching).^38^ The peaks at 1631.27 and 1027.67 cm^–1^ corresponded to the stretching of C=C and C–N bonds, indicating the presence of alkene and amines.^39^*Bathua* microgreens (Figure 4) showed a characteristic peak at 1625.67 cm^–1^, which was attributed to alkenes (C=C stretching), and a peak at 616.30 cm^–1^, indicating the presence of halo compounds (C–Br stretching), similar to the peaks obtained for *T. tinctoria* and *A. albicans* in a previous study. Bengal gram microgreens (Figure 4) showed peaks at 2917.04 and 2849.58 cm^–1^, indicating the presence of alkanes (C–H stretching), and a peak at 1636.11 cm^–1^, indicating the presence of alkenes (C=C stretching). The characteristic peak at 610.05 cm^–1^ indicated the presence of halo compounds (C–Br stretching).^36^

#### 3.4.4 Particle Size Distribution

**Figure 5:**
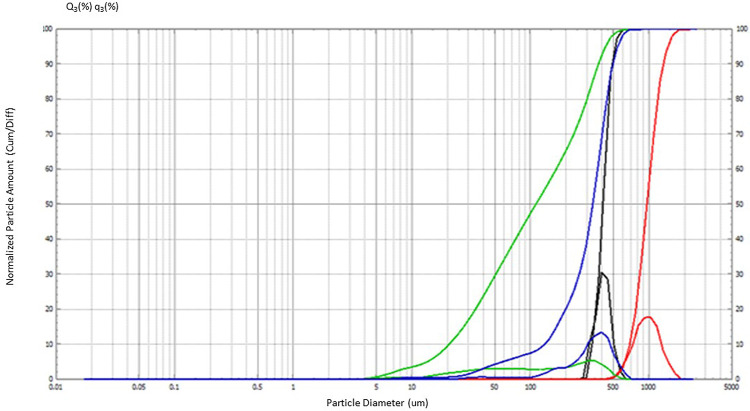
Representation of PSD of spinach (blue), carrot (black), *bathua* (red), and Bengal gram (green).

The study investigated the PSD of four different microgreens. The particle diameter ranged between 42.52 and 1341.39 μm (Figure 5). The powders that consisted of fine particles smaller than 100 μm exhibited high resistance to flow due to the cohesion between them.^33^ The PSD data are summarized in Table 4 for each microgreen. Spinach and carrot microgreens had similar maximum and minimum particle diameter values. For spinach microgreens, 90% of the particles had a maximum diameter of 498.82 μm, and 25% of the particles had a minimum diameter of 229.17 μm, with median, modal, and mean values of 330.20, 421.25, and 276.50 μm, respectively. In contrast, for carrot microgreens, 90% of the particles had a maximum diameter of 495.88 μm. *Bathua* had the highest median, modal, and mean values of 952.39, 857.77, and 949.10 μm, respectively, with a diameter ranging from 1341.39 to 796.19 μm among other microgreens. The lowest values for median (110.32 μm), modal (331.06 μm), and mean (97.56 μm) were found in Bengal gram with 90% of the particles having a maximum diameter of 370.40 μm, and 25% of the particles having a minimum diameter of 42.52 μm, respectively. Fine particle size showed higher wettability time due to low porosity and interspace voids, and further had higher water solubility and WHC.^34^

**Table 4:** PSD of Microgreens Depicting (Median, Modal, and Mean) and Maximum and Minimum Diameter in μm^a^

parameters	spinach	carrot	*bathua*	Bengal gram
median D (μm)	330.20 ± 0.51^c^	407.13 ± 1.69^b^	952.39 ± 0.81^a^	110.32 ± 0.47^d^
modal D (μm)	421.25 ± 1.70^b^	421.92 ± 1.71^b^	857.77 ± 0.81^a^	331.06 ± 0.47^c^
mean *V* (μm)	276.50 ± 1.7^c^	406.03 ± 2.40^b^	949.10 ± 1.2^a^	97.56 ± 1.24^d^
diameter (μm)				
maximum	498.82 ± 0.80^bc^	495.88 ± 1.24^b^	1341.39 ± 0.81^a^	370.40 ± 1.24^d^
minimum	229.17 ± 0.94^c^	362.53 ± 0.47^b^	796.19 ± 0.81^a^	42.52 ± 0.81^d^

a Values are means ± SD of triplicate analysis. Means with different letters in the same row indicate significant differences at *p <* 0.05.

### 3.5 Thermal Properties

#### 3.5.1 Differential Scanning Calorimetry

**Figure 6:**
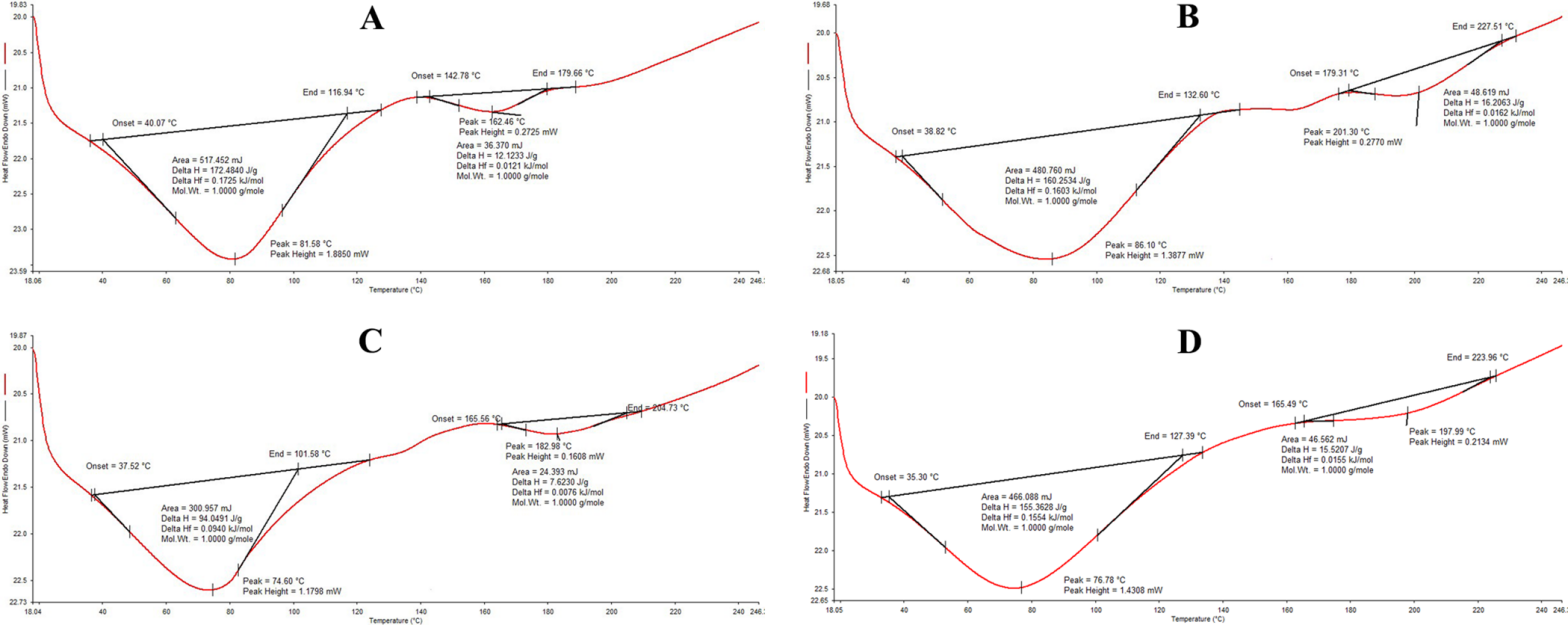
Typical DSC thermogram of (A) spinach, (B) carrot, (C) *bathua*, and (D) Bengal gram.

In this study, the thermal properties of spinach (*Spinacia oleracea*), carrot (*Daucus carota*), *bathua* (*C. album*), and Bengal gram (*Cicer arietinum*) microgreens were determined for the first time using DSC. The thermograms of the four microgreens are shown in Figure 6 and their endothermic peaks at different temperatures are discussed in Table 5. All the microgreens in the study showed endothermic reactions. In spinach microgreens (Figure 6), two endothermic peaks with onset temperatures of 40.07 and 142.78 °C and end set temperatures of 116.94 and 179.66 for peak 1 and peak 2, respectively, were observed. The denaturation of proteins resulted in the endothermic peaks at 81.58 and 162.26 °C.

**Table 5:** Determination of Thermal Behavior of Microgreens Using DSC

parameters	peaks	spinach	carrot	*bathua*	Bengal gram
peak temperature (°C)	1	81.58 ± 0.06^b^	74.60 ± 0.14^d^	76.78 ± 0.50^c^	86.10 ± 0.82^a^
	2	162.26 ± 0.12^d^	182.98 ± 0.70^c^	197.99 ± 0.28^b^	201.30 ± 0.50^a^
onset temperature (°C)	1	40.07 ± 0.82^a^	37.52 ± 0.2^c^	35.30 ± 0.56^d^	38.82 ± 0.37^b^
	2	142.78 ± 0.56^d^	165.56 ± 0.28^bc^	165.49 ± 0.12^b^	179.31 ± 0.15^a^
end set temperature (°C)	1	116.94 ± 0.74^c^	101.58 ± 0.37^d^	127.39 ± 0.45^b^	132.60 ± 0.22^a^
	2	179.66 ± 0.66^d^	204.73 ± 0.14^c^	223.96 ± 0.25^b^	227.51 ± 0.14^a^
enthalpy (J/g)	1	172.48 ± 0.45^a^	94.04 ± 0.23^d^	155.36 ± 0.21^c^	160.25 ± 0.13^b^
	2	12.12 ± 0.13^c^	7.62 ± 0.07^d^	15.52 ± 0.07^b^	16.20 ± 0.21^a^

Values are means ± SD of triplicate analysis. Means with different letters in the same row indicate significant differences at *p <* 0.05.

The thermal properties can indicate the extent of tertiary protein conformation.^40,41^ The denaturation of intramolecular bonds is an endothermic process.^42^ In carrot microgreens (Figure 6), the thermogram depicted an endothermic reaction that started at 37.52 °C and ended at 101.58 °C. The second endothermic peak started at 165.56 °C and ended at 204.73 °C. The broad peak at 74.60 °C indicated the degradation of some compounds, whereas the peak at 182.98 °C signified the denaturation or degradation of amines, carbohydrates, and lipids to some extent.^42^ The thermogram of *bathua* microgreens (Figure 6) showed two endothermic peaks at 74.60 and 182.98 °C, with the reaction starting from 35.30 °C and ending at 223.96 °C. The higher degradation at 74.60 °C may correspond to the degradation of some proteins, carbohydrates, and presence of some aromatic components (esters) in the sample. The low temperature of gelatinization of starch corresponds to the lower energy requirement for the initiation of starch gelatinization.^44^ The thermogram of Bengal gram microgreens (Figure 6) showed a broad peak at 86.10 °C with an onset temperature of 38.82 °C and an end set temperature of 132.60 °C, whereas the second peak at 201.30 °C indicated an onset temperature of 179.31 °C and an end set temperature of 227.51 °C. The denaturation of amines or carbohydrates might have occurred resulting in the observed endothermic peaks.^42^

## 4 Conclusions

The techno-functional and functional properties of Bengal gram and *bathua* microgreens were investigated in this study, revealing their potential applications in the food industry. The study uncovered *bathua*’s emulsifying and foaming properties, indicating its suitability as an ingredient in bakery products such as tarts and cakes, while Bengal gram exhibited potential for use in smoothies and juices, which is due to the suitable range of particle size of microgreen powder. Additionally, the microgreens’ antioxidant content suggested potential health benefits. SEM analysis revealed irregular and asymmetrical structures with creases and folds in the micrographs of the sample. FTIR spectroscopy identified the presence of alkanes, alkenes, alcohols, amines, phenols, and halo compounds, while XRD analysis indicated that the microgreens were amorphous, with wide and intense peaks. While this study provides valuable insights, further research is necessary to fully explore the potential of these microgreens in the food industry.
